# Depression and suicide risk during the Covid-19 pandemic at a Brazilian public health psychosocial addiction care center: a preliminary report

**DOI:** 10.47626/2237-6089-2021-0259

**Published:** 2022-05-26

**Authors:** Patricia Tejera de Moura, Camila Auth Rockenbach, Caroline da Rosa Mendes, Giovani Unterberger Mendes, Letícia Abruzzi Ghiggi, Marciane Diel, Patrícia Martini, Plauto Camozzato, Raquel Scafuto Barbosa de Castro, Rita Mello de Mello, Rossana Kovalski, Vauto Alves Mendes, Bruno Paz Mosqueiro

**Affiliations:** 1 Grupo Hospitalar Conceição Porto Alegre RS Brazil Grupo Hospitalar Conceição (GHC), Porto Alegre , RS , Brazil .

**Keywords:** Covid-19, depression, suicide, public health, alcohol, drugs

## Abstract

**Objective:**

To evaluate the impact of the Covid-19 pandemic on depressive symptoms and suicide risk among patients receiving treatment at a Public Health Psychosocial Addiction Care Center (CAPS AD III) in Porto Alegre, Brazil.

**Methods:**

Questions from the Coronavirus Health Impact Survey (CRISIS) translated into Brazilian Portuguese were used to evaluate 70 patients’ perceptions of and behaviors during the Covid-19 pandemic. Validated Brazilian versions of the Patient Health Questionnaire (PHQ-9) and the General Anxiety Disorder-7 (GAD-7) were used to evaluate the severity of depressive symptoms, suicide risk, and anxiety symptoms. A multiple logistic regression model was used to evaluate predictors of suicide risk in the sample.

**Results:**

Around 70% of patients reported moderate depressive symptoms and 30% reported severe depressive symptoms, 17% of patients reported having thoughts of suicide or death on more than half of days and 10% reported having them daily. The logistic regression model identified history of alcohol use as the main predictor of suicide risk in (OR 13.0, p = 0.03).

**Conclusions:**

Individuals with a history of alcohol consumption had significantly higher suicide risk scores at a psychosocial public health care center in Brazil during the Covid-19 pandemic. This result may be important for devising better strategies and interventions to support this specific population profile.

## Introduction

Since the first months of the year 2020, the world has witnessed rapid growth in infections caused by the coronavirus (COVID-19), which was officially characterized as a pandemic by the World Health Organization on March 11, 2020. ^1^ During this period, many studies have been carried out to assess the impacts of the Coronavirus pandemic on mental health. ^2 , 3^ Factors such as low socioeconomic status and presence of previous psychiatric illnesses have been associated with worse mental health outcomes in this period among health workers and in the general population. ^2 , 3^ However, few studies have evaluated the impact of the Coronavirus pandemic on more severe psychiatric patients, especially those treated at public services.

The effects of the Coronavirus pandemic may be more relevant when considering populations with even greater social vulnerability, including patients with alcohol and other types of substance use disorders. ^4 - 7^ Current aggravating factors include financial and economic difficulties, interpersonal conflicts, and domestic violence in some cases, as well as reduced access to care and support services such as therapeutic groups and elective consultations in many health services. ^8 - 10^

In a recent international position paper, specialists from different health areas reinforced the urgency of taking actions and conducting research to understand the impact of the pandemic and of isolation on people undergoing mental health treatment, as well as on people with serious mental disorders. ^11^

Thus, the present study aims to present an assessment carried out during the Covid-19 pandemic of depressive symptoms and risk of suicide in patients being monitored at a Psychosocial Care Center for Alcohol and Drugs (CAPS AD III) in Porto Alegre, RS, Brazil. To the best of our knowledge, this is the first report of potential implications of the Covid-19 pandemic for substance use disorder patients at a community-based addiction treatment service in Brazil. The results may be important for devising better strategies and interventions to support this population, especially in periods like the pandemic. ^12^

## Methods

The present study was a cross-sectional observational study of the prevalence and correlates of depressive symptoms and suicide risk in a sample of substance use disorder patients at a CAPS AD III in Porto Alegre, Brazil, during the first months of the Covid-19 pandemic. The study consecutively enrolled all adult patients who accepted the invitation to participate over a 12-week period from June to September of 2020. The inclusion criteria include being older than 18 years and presenting with diagnostic criteria for alcohol or other substance use disorder. Researchers were available for invitations and interviews for at least one hour a day during different work shifts at the CAPS AD III, in order to enroll individuals visiting the service at different times during the week. Informed consent was obtained from all individuals, in accordance with the terms of the approval granted by the institutional ethical committee. ^1^ A structured clinical questionnaire was developed to specifically address the impact of Coronavirus social distancing measures on addiction treatment, craving, substance use patterns, and socioeconomic variables. Validated Brazilian versions of the Patient Health Questionnaire (PHQ-9) and the General Anxiety Disorder-7 (GAD-7) instrument were used to evaluate the severity of depressive symptoms, suicide risk, and anxiety symptoms. Questions from the Coronavirus Health Impact Survey (CRISIS), ^13^ translated into Brazilian Portuguese, were used to evaluate patients’ perceptions of and behaviors during the Covid-19 pandemic (translation of the instrument into Brazilian Portuguese was conducted by Arthur G. Manfro, Danielle Soares, and Giovanni A. Salum, from Hospital de Clínicas de Porto Alegre, Universidade Federal do Rio Grande do Sul, Brazil). Individuals with cognitive impairments or severe psychiatric symptoms that limited their ability to comprehend the instrument or with acute intoxication (report of use 12 hours before the evaluation) were excluded from the study. A total of 80 individuals were invited to participate, 4 refused, 3 presented with acute intoxication or acute mental health distress, and 3 were unable to understand and answer the questionnaires. Descriptive analysis provided an overview of the patients’ sociodemographic and clinical characteristics, including their perceptions regarding the Coronavirus pandemic. A multiple logistic regression model was performed to evaluate predictors of higher suicide risk in the sample (item 9 of the PHQ-9 ≥ 2).

## Results

Most of the patients interviewed were male (66.7%), white (65.2%), separated (44.9%), and mostly unemployed (44.9%) or on leave of absence (14.4%). Less than half of the patients reported having already received a BRL 600.00 emergency aid payment from the government during the pandemic period (44.9%). Considering all patients, alcohol (73.9%) and cocaine (63.8%) were reported as the main substances used, followed by cannabis (36.2%) and crack (24.6%). More specifically, 58% of the patients reported consumption of multiple substances (n = 41). Most patients reported using alcohol or substances daily or almost daily in the last month (50.7%). Nearly a third of patients (34.7%) reported abstinence for more than one month. Approximately half of the patients identified an increase in cravings to use alcohol or substances during the Covid-19 pandemic (46.7%). In a specific analysis of the types of substances, 31% of alcohol users, 19% of marijuana users, 30% of cocaine users, and 15.4% of crack users reported an increase in consumption of these substances during the pandemic (Tables 1 and 2).

In addition, 81.2% of patients reported seeking care at the CAPS AD III due to worsening of mental health symptoms and 8.8% reported seeking care at psychiatric emergency services due to mental health crisis situations.

Regarding patients’ perceptions about the pandemic period, 34.7% reported being very or extremely concerned about a possible Coronavirus infection and 36.2% of patients reported reading and talking a lot about the topic. Most individuals reported little time devoted to outdoor activities (63.7%), no physical activity (53.6%), and much less contact with people outside the home (81.1%). The majority (62.2%) of individuals reported financial difficulties and 44.9% had concerns about housing, while 53.6% of patients expressed concerns about going hungry due to lack of money during the pandemic ( Table 1 [Table t2] ).

**Table 1:** Sociodemographic and clinical variables of substance use disorder patients from a psychosocial addiction care center in Brazil (n = 70)

Variable	n	%
Male	46	66.7
White	45	65.2
Non-White	25	34.8
Single	9	13.0
Married	28	40.6
Divorced	31	44.9
Widowed	1	1.4
Employed	20	29.0
Unemployed	31	44.9
Retired	7	10.1
Health insurance	10	14.4
Receiving temporary government financial support (BRL 600.00)	31	44.9
Substances		
Alcohol	51	73.9
Cocaine	44	63.8
Crack-Cocaine	17	24.6
Cannabis	25	36.2
Benzodiazepines	3	4.3
Tobacco	24	34.8
Other	9	13.0
Frequency of use of the main substance (last month)		
1-3 times a month	4	5.8
1-2 times a week	6	8.7
3-6 times a week	12	17.4
Daily	23	33.3
Abstinent more than a month	24	34.7
Increased craving during the Covid-19 pandemic	32	46.7
Increased alcohol consumption	18	31.0
Increased cannabis consumption	8	19.0
Increased cocaine consumption	15	30.0
Increased crack-cocaine consumption	6	15.4
Sought mental health consultations at the CAPS AD	56	81.2
Sought psychiatric emergency consultations	13	18.8
Sought health care services because of coronavirus suspicion	13	19.1
Depression diagnosis according to PHQ-9*	48	69.6
Severe depressive symptoms according to PHQ-9*	21	30.4
Suicide risk according to PHQ-9 ^†^		
Never	41	59.4
Several days	9	13.0
More than half of the days	12	17.4
Almost every day	7	10.1

BRL = Brazilian *reais* ; PHQ-9 = Patient Health Questionnaire.

* PHQ-9 ≥ 10 (depression), PHQ-9 ≥ 20 (severe depression).

^†^ PHQ-9 item 9: Over the last two weeks, thoughts that you would be better off dead, or of hurting yourself in some way?

**Table 2 t2:** Participants’ answers to PHQ-9 questions

Question	Answer categories				
During the past two weeks:	Not at all	Slightly	Moderately	Very	Extremely
Worried about being infected	27.5	21.7	15.9	21.7	13.0
Worried about family or friends being infected	11.6	21.7	13.0	40.6	13.0
Worried about physical health being affected	21.7	30.7	7.2	23.2	17.4
Worried about mental health being affected	18.8	24.6	13.0	21.7	21.7
Extent of reading or talking about coronavirus	15.9	31.9	15.9	30.4	5.8
	**Not at all**	**1-2 days**	**A few days**	**Several days**	**Every day**
Time spent outside of the home per week	15.9	27.5	20.3	18.8	17.4
	**None**	**1-2 days**	**3-4 days**	**5-6 days**	**Every day**
Time spent doing exercise per week (> 30 min a day)	53.6	20.3	7.2	8.7	10.1
During the past two weeks:	**A lot less**	**A little less**	**The same**	**A little more**	**A lot more**
Extent of contact with people outside home	47.8	33.3	13.3	4.3	1.4
During the past two weeks:	**A lot worse**	**A little worse**	**The same**	**A little better**	**A lot better**
Changes in the quality of family relationships	5.8	20.3	49.3	13.0	11.6
	**Not at all**	**Slightly**	**Moderately**	**Very**	**Extremely**
Financial problems related to Coronavirus pandemic	15.9	21.7	7.2	36.2	18.8
Concerns about stability of living situation (housing)	42.0	13.0	10.1	23.2	11.6
Hopes the pandemic will end soon	15.9	20.3	18.8	29.0	15.9
	**Yes**			**No**	
Worry about running out of food because of lack of money	53.6			44.9	

PHQ-9 = Patient Health Questionnaire.

Of the patients interviewed, 69.6% had at least moderate depressive symptoms, and 30.4% had severe symptoms (PHQ-9 > 20). Regarding thoughts of death and suicidal ideation, 59.4% reported no such symptoms, 13% reported having such thoughts on a few days in the last 2 weeks, 17.4% on more than half of the days, and 10% almost daily ( Table 1 ).

The logistic regression analysis identified alcohol use (OR 13.0, p = 0.03) and greater anxiety symptoms measured using the GAD-7 scale (OR 1.1, p = 0.02) as the main predictors of suicide risk. Sex, income, and use of other substances such as cannabis, cocaine, and crack-cocaine did not have statistically significant predictive effects for increased risk of suicide in the model studied.

## Discussion

In a sample of patients undergoing treatment for substance use disorders at a CAPS AD III, most reported at least moderate depressive symptoms (70%) and about 30% had criteria for severe depression. Even more relevant, 10% reported serious risk of suicide with daily thoughts of death or suicide. In addition, history of alcohol use was identified as the main predictor of suicide risk during the Coronavirus pandemic. This is an original study, which is relevant for understanding the impact of the pandemic on a sample of individuals with severe mental disorders and little access to participation in other epidemiological studies conducted through online surveys.

The results are in line with findings showing that patients with a history of mental disorders are suffering from higher levels of depressive symptoms during the current pandemic. ^3 , 14^ In addition, previous studies confirm that individuals with disorders related to use of alcohol and drugs have a higher prevalence of depressive symptoms. In the last Brazilian National Survey of Alcohol and Drugs (LENAD-II), for instance, about 25% of people in the general population reported symptoms of depression, compared to 41% of alcohol users, 30% of marijuana users, and 42% of cocaine users. ^15^ Nonetheless, it is not possible to confirm that there has been an increase in the incidence or severity of symptoms of depression due to the lack of previous data for comparison from the same population investigated in our study.

A systematic review in June 2020 identified 41 studies measuring levels of depression during the Covid-19 pandemic. Of these, 14 studies addressed the general population with prevalence between 22% and 33%, and three studies evaluated the prevalence among patients diagnosed with Covid-19, with results between 48% and 62%. It is important to note that only one study evaluated patients with psychiatric diseases. ^2^ Another systematic review in the same period identified the presence of depressive symptoms in between 14.6% and 48.3% of the general population during the pandemic. ^3^

In the present study, approximately half of the patients (46.7%) identified an increase in cravings to use alcohol or substances and many individuals (15-30%) reported an increase in substance use during the pandemic. Furthermore, a significant proportion of patients (62.2%) mentioned financial difficulties, concerns about housing (44.9%), and concerns about going hungry (53.6%). Activities with potentially protective effects, including social contacts and physical activity were significantly reduced.

Present to some extent in 40.4% of patients, risk of suicide is another factor of great concern that was considered in our study. According to data from LENAD-II, the prevalence of suicidal ideation was 20.8% in alcohol users, 31.5% in cannabis users, and 40% in cocaine users. Suicide attempts were identified in 12.4% of problem alcohol users, 16.5% of cannabis users, and 20.8% of cocaine users. ^16^ An interesting aspect found in our study is that risk of suicide was more strongly associated with alcohol consumption than with other substances. It is certainly important to understand how the pandemic and social distancing measures might have affected alcohol and substance use patterns in substance use disorder patients attending the CAPS AD. Most patients (63.7%) reported a significant decrease in their time devoted to outdoor activities during the pandemic. The increased amount of alcohol consumption (reported by one third of our sample) might have been a strategy used by many individuals to deal with isolation, uncertainty, fear, and other sources of conflicts and distress and should be considered as a potential mechanism for understanding the relationship between alcohol and suicide risk. Harmful alcohol use and alcohol use disorders are definitely one of the main risk factors for suicidal behavior and may well be a significant risk factor for suicide. ^17^ A recent survey in the UK, for instance, identified increased alcohol consumption from before to during the lockdown, and this was independently associated with poor mental health and depressive symptoms. ^7^ The importance of measuring drug and alcohol consumption patterns and suicidal behavior rates in the current context is evident and they may not only rise during the pandemic, but may also remain high for a long period and have a late peak. ^18 , 19^

The findings must be considered taking the study’s limitations into account. Firstly, this is a preliminary study with a cross-sectional design, which prevents causal inferences between the variables studied. Our sample included substance use disorder patients attending a secondary care addiction psychosocial treatment setting who need mental health care during the pandemic. Thus, the association between alcohol and suicide could not be directly generalized to all substance use disorder patients or the general population. However, considering the specificities of the sample of patients, which are difficult to access in other epidemiological surveys, the study makes an important contribution to understanding the impact of the Coronavirus pandemic, especially regarding the risk of suicide in vulnerable individuals with more serious mental disorders.

## References

[1] . World Health Organization (WHO). Timeline: WHO’s COVID-19 response [Internet]. [cited 2021 Sep 8]. www.who.int/emergencies/diseases/novel-coronavirus-2019/interactive-timeline#!

[2] . Luo M, Guo L, Yu M, Jiang W, Wang H. The psychological and mental impact of coronavirus disease 2019 (COVID- 19) on medical staff and general public – a systematic review and meta-analysis. Psychiatry Res. 2020;291:113190. 10.1016/j.psychres.2020.113190 PMC7276119 32563745

[3] . Xiong J, Lipsitz O, Nasri F, Lui LM, Gill H, Phan L, et al. Impact of COVID-19 pandemic on mental health in the general population: a systematic review. J Affect Disord. 2020;277:55-64. 10.1016/j.jad.2020.08.001 PMC7413844 32799105

[4] . Karamouzian M, Johnson C, Kerr T. Public health messaging and harm reduction in the time of COVID-19. Lancet Psychiatry. 2020;7:390-1. 10.1016/S2215-0366(20)30144-9 PMC7185931 32353270

[5] . Kelley M, Ferrand RA, Muraya K, Chigudu S, Molyneux S, Pai M, et al. An appeal for practical social justice in the COVID-19 global response in low-income and middle-income countries. Lancet Glob Health. 2020;8:E888-9. 10.1016/S2214-109X(20)30249-7 PMC7255203 32416766

[6] . Zaleski M, Laranjeira RR, Ratto L, Romano M, Soares MB, Abelardino V, et al. Guidelines of the Brazilian Association of Studies on Alcohol and Other Drugs (ABEAD) for diagnoses and treatment of psychiatric comorbidity with alcohol and other drugs dependence. Braz J Psychiatry. 2006;28:142-8. 10.1590/s1516-44462006000200013 16810399

[7] . Jacob L, Smith L, Armstrong NC, Yakkundi A, Barnett Y, Butler L, et al. Alcohol use and mental health during COVID-19 lockdown: a cross-sectional study in a sample of UK adults. Drug Alcohol Depend. 2021;219:108488. 10.1016/j.drugalcdep.2020.108488 PMC7768217 33383352

[8] . Fazel S, Runeson B. Suicide. N Engl J Med. 2020;382:266-74. 10.1056/NEJMra1902944 PMC7116087 31940700

[9] . Kawohl W, Nordt C. COVID-19, unemployment, and suicide. Lancet Psychiatry. 2020;7:389-90. 10.1016/S2215-0366(20)30141-3 PMC7185950 32353269

[10] . Ungar M, Theron L. Resilience and mental health: how multisystemic processes contribute to positive outcomes. Lancet Psychiatry. 2020;7:441-8. 10.1016/S2215-0366(19)30434-1 31806473

[11] . Holmes EA, O’Connor RC, Perry VH, Tracey I, Wessely S, Arseneault L, et al. Multidisciplinary research priorities for the COVID-19 pandemic: a call for action for mental health science. Lancet Psychiatry. 2020;7:547-60. 10.1016/S2215-0366(20)30168-1 PMC7159850 32304649

[12] . Yao H, Chen JH, Xu YF. Patients with mental health disorders in the COVID-19 epidemic. Lancet Psychiatry. 2020;7:e21. 10.1016/S2215-0366(20)30090-0 PMC7269717 32199510

[13] . Nikolaidis A, Paksarian D, Alexander L, Derosa J, Dunn J, Nielson D, et al. The Coronavirus Health and Impact Survey (CRISIS) reveals reproducible correlates of pandemic-related mood states across the Atlantic. Sci Rep. 2021;11:8139. 10.1038/s41598-021-87270-3 PMC8046981 33854103

[14] . Hao F, Tan W, Jiang L, Zhang L, Zhao X, Zou Y, et al. Do psychiatric patients experience more psychiatric symptoms during COVID-19 pandemic and lockdown? A case-control study with service and research implications for immunopsychiatry. Brain Behav Immun. 2020;87:100-6. 10.1016/j.bbi.2020.04.069 PMC7184991 32353518

[15] . Unidade de Pesquisas em Álcool e Drogas (UNIAD), Instituto Nacional de Ciência e Tecnologia para Políticas Públicas do Álcool e outras Drogas (INPAD). II LENAD - Segundo levantamento nacional de álcool e drogas [Internet]. 2012 [cited 2021 Sep 8]. inpad.org.br/wp-content/uploads/2014/03/Lenad-II-Relatório.pdf

[16] . Abdalla RR, Miguel C, Brietzke E, Caetano R, Laranjeira R, Madruga CS. Suicidal behavior among substance users: data from the Second Brazilian National Alcohol and Drug Survey (II BNADS). Braz J Psychiatry. 2019;41:437-40. 10.1590/1516-4446-2018-0054 PMC6796807 30785535

[17] . Borges G, Bagge CL, Cherpitel CJ, Conner KR, Orozco R, Rossow I. A meta-analysis of acute use of alcohol and the risk of suicide attempt. Psychol Med. 2017;47:949-57. 10.1017/S0033291716002841 PMC5340592 27928972

[18] . Sher L. The impact of the COVID-19 pandemic on suicide rates. QJM. 2020;113:707-12. 10.1093/qjmed/hcaa202 PMC7313777 32539153

[19] . Gunnell D, Appleby L, Arensman E, Hawton K, John A, Kapur N, et al. Suicide risk and prevention during the COVID-19 pandemic. Lancet Psychiatry. 2020;7:468-71. 10.1016/S2215-0366(20)30171-1 PMC7173821 32330430

